# Effects of Top-hat Laser Beam Processing and Scanning Strategies in Laser Micro-Structuring

**DOI:** 10.3390/mi11020221

**Published:** 2020-02-20

**Authors:** Hoang Le, Pavel Penchev, Anne Henrottin, David Bruneel, Vahid Nasrollahi, Jose A. Ramos-de-Campos, Stefan Dimov

**Affiliations:** 1 Department of Mechanical Engineering, University of Birmingham, Birmingham B15 2TT, UK; p.penchev@bham.ac.uk (P.P.); v.nasrollahi@bham.ac.uk (V.N.); s.s.dimov@bham.ac.uk (S.D.); 2 LASEA, Rue des Chasseurs Ardennais 10, 4031 Angleur, Belgium; ahenrottin@lasea.com (A.H.); dbruneel@lasea.com (D.B.); jaramos@lasea.com (J.A.R.-d.-C.)

**Keywords:** beam shapers, top-hat laser beam, gaussian laser beam, micro-structuring, pulse energy, hatch distance, pulse distance

## Abstract

The uniform energy distribution of top-hat laser beams is a very attractive property that can offer some advantages compared to Gaussian beams. Especially, the desired intensity distribution can be achieved at the laser spot through energy redistribution across the beam spatial profile and, thus, to minimize and even eliminate some inherent shortcomings in laser micro-processing. This paper reports an empirical study that investigates the effects of top-hat beam processing in micro-structuring and compares the results with those obtainable with a conventional Gaussian beam. In particular, a refractive field mapping beam shaper was used to obtain a top-hat profile and the effects of different scanning strategies, pulse energy settings, and accumulated fluence, i.e., hatch and pulse distances, were investigated. In general, the top-hat laser processing led to improvements in surface and structuring quality. Especially, the taper angle was reduced while the surface roughness and edge definition were also improved compared to structures produced with Gaussian beams. A further decrease of the taper angle was achieved by combining hatching with some outlining beam passes. The scanning strategies with only outlining beam passes led to very high ablation rates but in expense of structuring quality. Improvements in surface roughness were obtained with a wide range of pulse energies and pulse and hatch distances when top-hat laser processing was used.

## 1. Introduction

Direct laser micro-structuring is an attractive solution for fabricating micro-scale features due to advantages offered in overcoming some material and dimensional limitations of conventional machining methods. In particular, the inherent capabilities of laser structuring, such as non-contact processing, relatively high ablation rates at micro-scale, good accuracy, and repeatability, makes it a very attractive proposition compared with other competing micro-processing technologies, e.g., mechanical machining and lithography.

Currently, the majority of commercially available laser micro-processing systems employs Gaussian beams (TEM_00_) due to their intensity profile and their consistency along propagation direction. This makes Gaussian beams much easier to use and calibrate. However, their spatial intensity distribution leads to an energy waste at the “tails” of the beam profiles as the intensity is lower than ablation thresholds and is just sufficient to melt/heat the material. Thus, this increases the heat affected zone (HAZ) while decreasing the processing efficiency of delivered pulse energies. Therefore, an energy redistribution across the beam spatial profile through the use of beam shapers can minimize and even avoid the pulse tail and, thus, achieving the desired intensity distribution at the laser spot [1]. In particular, Gaussian spatial intensity can be transformed into top-hat, donut, or reverse Gaussian distribution by employing beam shapers.

In this context, a uniform spatial intensity, i.e., a top-hat beam profile, can improve the processing condition at the laser spot and lead to higher geometrical accuracy and repeatability, lower surface roughness, and higher laser micro-structuring quality in general. Especially, the top-hat intensity profiles can minimize the recast area, i.e., the splashes of material at the edges of the structures by reducing HAZ, as most of the energy in the irradiance is over the ablation threshold. In addition, the top-hat beam is expected to improve the resulting surface roughness and reduce taper angles due to the sharp intensity gradient at the pulse tails.

Thus, the objective of this research is the investigation of effects of top-hat beams in laser micro-structuring and compare the resulting structures with those achievable with Gaussian beams. The quality of the structures is studied in terms of HAZ, geometrical accuracy, i.e., taper angle, and surface roughness, while the processing efficiency is considered, too. In addition, the effects of different scanning strategy on structure quality and processing efficiency are analyzed in this research.

## 2. Literature Review of Laser Beam Shaping Application

### 2.1. Gaussian Beam Processing

Gaussian beams have high peak power and if a suitable wavelength is selected any materials can be processed with acceptable quality of resulting structures. Gaussian beams are self-consistent and, thus, the intensity profile is maintained at the same level along the propagation direction and practically beam waist and lens focal plane can be considered coincidental [2]. These attractive properties make them reliable and easy to use and therefore they are widely deployed in many laser processing applications [3]. However, Gaussian distribution partly contributes to the inherent shortcoming of laser micro-machining processes such as tapering effects at side walls and HAZ on the machined parts [4,5]. The tapering effects are present after most of laser processing applications with Gaussian beams, e.g., drilling [6,7] or micro-structuring (trenches, 3D structures) [8,9]. Such draft angles at side walls affect the geometric accuracy of produced structures and also can impact the functional performance.

The tapering effects of structures can be explained with non-uniform energy distribution of a Gaussian beam that entails non-uniform ablation. The high peak power of Gaussian profiles leads to high ablation rates within only a small area at the center of the beam spots and lower ablation rates (or no ablation) at their surroundings. Thus, it is not possible to have uniform laser-material interactions across the beam spots. This leads to non-uniform laser ablation and formations of recasts when a structures’ depth increases, i.e., due to difficulties in evacuating the ablated material, especially in percussion drilling [7]. In addition, there are some other negative side effects in Gaussian beam processing on produced structures such as increased roughness.

At the same time, the demand together with requirements for high accuracy machining are constantly increasing. Furthermore, there is a consistent trend for miniaturization of new and existing devices that drives the advances in laser micro-machining. Efforts to achieve high geometrical accuracy, especially to minimize the tapering effect on side walls of structures fabricated by laser micro-machining, was the focus of many investigations, and different approaches to address this issue were suggested by researchers [10,11,12,13,14]. The main difficulties with these solutions are either the high accuracy requirements for the movements of mechanical stages, or their low repeatability and also their applicability for only specific structures. Laser micro-drilling of holes under flowing water was tried by Wee et al. and compared to those produced in air [15]. Water acted as a coolant and also removed the debris from the processed area and, thus, cleaner holes were produced with smaller draft angles.

### 2.2. Laser Beam Shaping Technology

Beam shapers was considered as an effective generic solution for minimizing the tapering effect because it did not require complex multi-axis processing strategies and at the same time it was relatively easy to integrate into existing laser processing systems. In addition, it was judged that the beam shaping technology led to improvements in laser processing efficiency [16] and quality of produced structures, too.

The latest beam shaping solutions fall into two main groups [1,17,18,19,20,21,22], i.e., multifaceted beam integrators based on reflective phase elements and field mapping employing diffractive and refractive optical element. The multifaceted beam integrators can be of different types, e.g., micro-lens arrays, spatial light modulator (SLM), or multi-plane light conversion (MPLC). By employing MPLC based light modulators, laser light can be split and recombined to create different complex shapes such as linearly polarized (LP) modes [23,24]. A combination of MPLC based light modulators with binary masks was deployed successfully to create complex laser beam shapes [25]. Hafner et al. used liquid crystal on silicon for spatial light modulation (LCoS-SLM) and acousto-optic beam shaping (AOS) to generate flat-top laser profiles that employed them for accurate laser micro-machining [24]. The main shortcoming of beam shapers that involve laser beam splitting and recombining is that they affect the beam consistency and process efficiency and, thus, can be significant drawbacks in laser micro-machining.

At the same time, the field mapping technology employs complex surface optic elements to remap the energy distribution of Gaussian beams in a controlled manner and, thus, achieving a variety of desirable profiles, e.g., such as donut, Airy, top-hat, or reverse Gaussian. There are different types of field mapping beam shapers, e.g., single optic field mappers and dual optic field mappers [26]. An important advantage of the field mapping technology is that it can deliver collimated and low divergence output beams [27]. In this way, the beam consistency can be maintained along the propagation direction and, thus, achieving high quality beam profiles at a given plane/distance.

Two types of optical elements are used in field mapping solutions, i.e., refractive optic elements (ROEs) and diffractive optic elements (DOEs). DOEs are typically thin and light weight and therefore the diffractive field mapping has a lower resistance to laser light and can also lead to energy losses [28]. At the same time, small optical elements allow compact beam shapers to be designed that are very suitable for integration in relatively small laser processing systems. The main disadvantage of DOE based field mapping is that resulting beam is very sensitive to any misalignments and the input quality of Gaussian beams. In particular, it was reported that a laser processing system with an integrated DOE based beam shaper to produce a flat-top beam required any angular and liner misalignments between beam and shaper axes to be less than 140 μrad and 25 μm, respectively [29]. This makes the installation and calibration procedures of DOE based field mapping solutions very time consuming and cumbersome.

The other field mapping solution employs ROEs to deflect each pixel on an input plane at a specific angle and, thus, creating a given profile at the image plane [28,30]. Some advantages of the refractive field mapping are its high transmission efficiency, a relatively simple design that make them easier to manufacture and also ROE based shaper are easier to integrate into existing laser processing systems [31]. In addition, it is important to stress that ROE based beam shapers have a bigger depth of focus, almost the same as that of Gaussian beams. This makes the setting up of any laser micro-processing operation much easier and at the same time it is a critical requirement in any 3D laser machining. In addition, refractive beam shapers are recommended when a beam spot size of less than 100 μm have be achieved in the focal plane [32].

### 2.3. Application of Top-Hat Beams in Laser Micro-Machining

The beam shaping technology can be applied to obtain different beam profiles, e.g., such as Bessel, donut (or annular), and top-hat beams, and, thus, addressing specific laser processing requirements [33,34,35,36,37]. The use of top-hat profiles to improve the resulting surface roughness and tapering, has gained a significant attention due to the uniform energy distribution and more efficient processing [38]. Especially, the top-hat laser micro-processing offers important advantages in micro-hole drilling [39], micro-channel scribing [40], and micro-structuring in general. Coutts et al. used nanosecond laser with top-hat profile to drill micro-holes on brass and ceramics substrates [41]. Their research has shown a significant improvement in holes’ quality, especially in drilling brass plates compared to a conventional Gaussian profile. In another investigation, a square top-hat beam was used to scribe 150 nm thin films of indium tin oxide using both refractive and diffractive beam shapers [42,43]. The uniform energy profile of the square top-hat beam allowed for a smaller pulse overlap to be used and this led to nine-fold increase in the scribing speed compared to Gaussian beam processing while achieving a similar quality. In general, the reported studies provided clear evidences about the potential improvements that top-hat processing can offer in laser micro-machining, in particular in minimizing the side wall taper and surface roughness while improving the processing efficiency.

The beneficial properties of top-hat laser beams were predominantly deployed in drilling micro-holes and also in scribing micro-channels. The sizes of machined holes and channels in these investigations were similar to that of the beam spot size and, thus, the effect of the uniform energy profile in the processing area can be seen clearly. However, micro-machining of large structures in regards to the beam spot size should be considered a different process that requires optimization both in processing strategies and process parameters in order to obtain improvements in processing performance [44]. Thus, it is important to investigate what improvements top-hat laser machining can offer when the technology is used for producing relatively large structures. In this context, this research is an attempt to address the gap in our knowledge about the capabilities that top-hat processing offers in laser micro-machining.

## 3. Methodology

### 3.1. Material

Silicon (Si) wafers with thickness of 500 µm and average surface roughness of approximately 27 nm were used in this empirical study. Laser structuring of silicon gives less recast and debris when compared with those resulting after processing of metal substrates, in particular around the structure edge. Therefore, the structures are usually much better defined and, thus, the measurement uncertainty can be minimized when inspecting them. So, the use of Si wafers provides a better condition to assess the effects of investigated beam intensity profiles. In addition, Si wafers are commonly used in a wide range of industrial applications, e.g., solar cells [45] and electronics circuits.

### 3.2. Laser Source and Beam Delivery System

The structuring experiments were carried out on LS5 LASEA system that integrates a MOPA-based Yb fiber laser with a nominal wavelength of approximately 1064 nm, average power up to 50 W and beam quality factor M^2^ ≤ 1.3. The pulse duration can be varied from 15 to 220 ns with a maximum pulse energy of 0.71 mJ. A nanosecond laser has been employed because it is widely used by industry for micro-structuring because it offers acceptable trade-offs between processing efficiency and a structures’ quality. The beam movements were controlled with a Rhothor RTA XY scan head with a positional resolution better than 8 µrad, repeatability less than 15 µrad, and scanning speed up to 2400 mm/s.

A telecentric lens with focal length of 100 mm was integrated in the laser setup that provided a spot size of approximately 50 µm at focal plane and depth of focus of approximately 2.6 mm. The relatively large depth of focus ensures that the top-hat profile is achieved within the effective ablation range of the lens. Furthermore, the beam profile changes, continuously, between top-hat, donut, and Airy profiles along the propagation direction [46]. Therefore, more than one top-hat profile may be obtained within the depth of focus and, thus, such beam delivery setups provide more flexibility. The use of a telecentric lens was essential in this research because the capabilities of a top-hat beam shaper was assessed for performing laser micro-processing operations. Especially, a constant beam path length from the lens to the workpiece surface was maintained and, thus, to have a consistent laser intensity profile at the focal plane, i.e., to keep top-hat profile at the beam waist.

### 3.3. Top-Hat Beam Shaper

The refractive field mapping method was employed to change the beam intensity profile from Gaussian to top-hat. This method was selected while taking into account advantages and disadvantages of different beam shaping technologies discussed in Section 2.2 and also the relatively easy integration of ROE based shapers into laser processing systems. An important advantage of the refractive field mapping method is that a top-hat beam can be achieved with a minimum energy loss and misalignment when compared with other methods such as beam truncation with an aperture or using an array of micro-lenses [21]. In addition, the sensitivity of the intensity profile to the shaper’s position along the beam path is much less compared with the diffractive field mapping method and this is an important consideration when retrofitting a shaper into existing laser processing systems.

A Focal-πShaper 9_1064 shaper with an optimum wavelength in the range from 1020 to 1100 nm was integrated into the beam delivery system before the XY scan head. The installation and calibration process of the Focal-π Shaper plays a very important role in obtaining good quality beam profile at the lens focal plane. Any small misalignment can contribute to low quality of the top-hat profile at the focal plane. Therefore, a precise alignment procedure was employed to correct the position of the beam shaper stand and, thus, to align it to the beam propagating direction.

**Figure 1:**
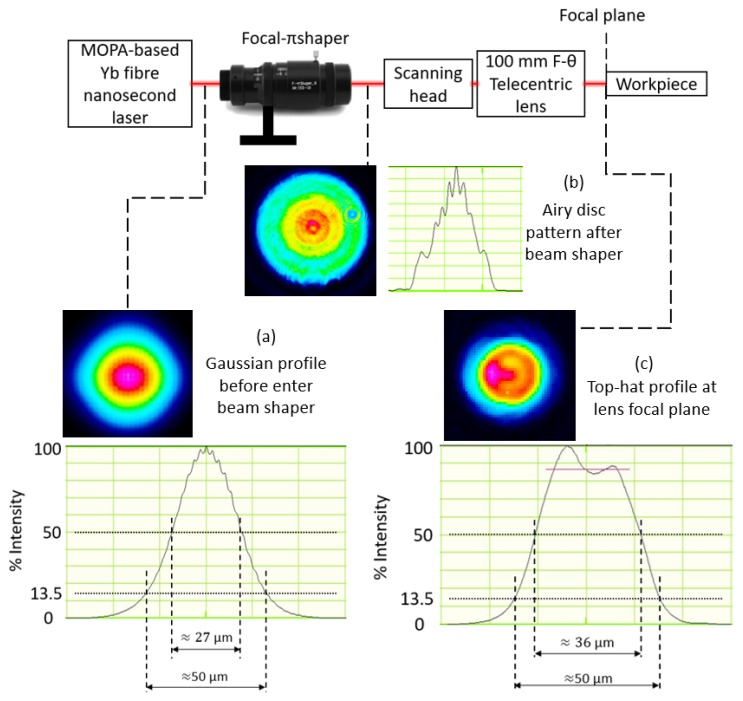
Intensity profiles at different planes along the beam propagation direction: (**a**) before and (**b**) after the beam shaper and (**c**) at the focal plane.

The beam profile at the different planes along the propagation direction was measured employing WinCamD-LCM–1” CMOS beam profiler. The input Gaussian beam was transformed into Airy profile, first, after passing through Focal-πShaper as a result of refractive field mapping. Then, through Fourier transformation the intensity profile was converted from Airy to top-hat at the focal plane as shown in Figure 1. This is in agreement with the theoretical Fourier transformation of Airy intensity profiles along the propagation direction [32,47]. The top-hat profile shown in Figure 1 is also the best top-hat that can be achieved within depth of focus of the lens. It is important to note that the laser power could decrease because of beam shaper integration into the beam delivery system. Therefore, the laser power was also measured after the focusing lens with laser power meter (Gentec UNO Laser Power Meter). The drop of laser power was compensated by increasing the average power and, thus, maintaining constant power levels during the experiments in this research. The spot sizes of both Gaussian and top-hat beams were kept approximately 50 μm at the focal plane.

### 3.4. Design of Experiment

Three sets of experiments were conducted to investigate the capabilities of top-hat laser processing and compare them with the structuring results achievable with Gaussian beam.

#### 3.4.1. Structuring with Different Processing Strategies

The structuring quality in this research was assessed based on resulting surface roughness, the tapering effect on the structures’ side wall, HAZ, and edge definition. Arrays of square pockets with dimension of 1.5 by 1.5 mm^2^ were produced with Gaussian and top-hat beams on Si wafers by applying the process settings in Table 1. The pulse distance (*P_d_*) was maintained the same, 10 µm, by adjusting pulse frequency (*f*) and/or scanning speed (*v*) by using the following equation:

*P_d_* = *v*/*f* (1)

**Figure 2:**
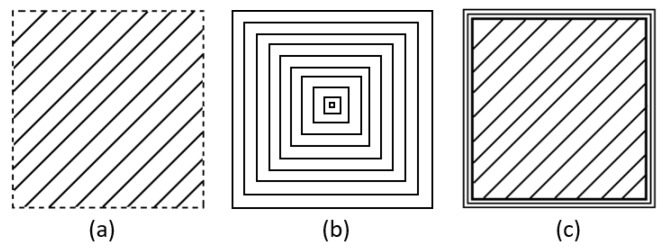
Laser scanning strategies: (**a**) hatching with 15 µm step over between the passes and 45 degrees shifts between the layers; (**b**) reduction outlining with 15 µm step over between the passes; and (**c**) a combination of (**a**,**b**).

The optimal hatch and pulse distances to achieve a good surface quality was determined after conducting some initial structuring trials. Three scanning strategies as depicted in Figure 2 were considered in producing the pockets layer by layer, i.e.,:

(a) Hatching with 45 degree angular shift between layers;
(b) Reduction outlining from outside to inside;
(c) A combination of hatching and five outlining passes after each layer.

The laser delays were optimized to achieve the best possible structuring quality [9]. Five pockets were produced with each strategy and the processing parameters in Table 1 and the average results were analyzed in this research. These parameters were chosen based on some initial trials. The focal plane is kept at the workpiece surface throughout the experiments.

#### 3.4.2. Structuring with Varying Hatching and Pulse Distances

The effects of top-hat profile on ablation rates, specially the pocket depth, and the resulting surface roughness on pocket bottoms were investigated by varying pulse and hatching distances. In particular, the pulse distance was varied in the range from 5 to 14.3 µm by increasing the scanning speed from 750 to 2150 mm/s while keeping the pulse frequency constant at 150 kHz. This helps to avoid the effect of frequency variation which is outside the scope of this study. At the same time, the hatching distance was varied from 5 to 25 µm with an increment of 5 µm as shown in Table 2. The structures were fabricated with the hatching (a) strategy, especially with only hatching passes, while other parameters were kept the same as given in Table 1. Both Gaussian and top-hat beams were used to compare the results. Five pockets were produced with each process setting and the average results were analyzed.

#### 3.4.3. Structuring with Varying Pulse Energies

Arrays of square pockets were produced with Gaussian and top-hat beams while pulse energies were varied in the range from 0.04 to 0.256 mJ. Especially, the pulse energies were varied by changing laser power settings while the pulse frequency was kept constant at 150 kHz. This set of experiments were conducted again with the first structuring strategy and also the process settings in Table 1 were used with a hatch distance of 15 μm. As in other experiments, five pockets were produced with each process setting and the average results were analyzed.

### 3.5. Inspection

The structured Si wafers were cleaned in an ultrasonic bath and then inspected with a focus variation microscope system (Alicona G5) and a scanning electron microscope (SEM) system (JEOL JCM-6000). All pockets in the three sets of experiments were scanned with the same resolution settings to obtain their 3D profiles by Alicona G5. The surface roughness was measured over a square area of 700 by 700 µm^2^ at the center of the pockets’ bottom that was smaller than the minimum required by the standard to measure the true roughness. However, the objective of this research was to compare the structuring performance of top-hat and Gaussian beams and therefore it was more important to keep measurement conditions identical.

The roughness was measured at the centers of fabricated structures to avoid any negative effects near the pockets’ edges, especially due to the side walls’ tapering, recasts, or beam deflection effect, which can lead to a lower surface quality. Such side effects may prevent judging correctly about the true effects of these two different beam profiles. The taper angles were considered the deviations of the side wall profiles from the normal to the structured planar surface. While, the HAZs were evaluated based on the resulting color changings along the structures’ edges.

## 4. Results and Discussion

### 4.1. Structuring with Different Processing Strategies

#### 4.1.1. Quality of Structures

The inspection results from structures produced with three different processing strategies are provided in Table 3. In general, the use of the top-hat beam led to lower surface roughness, i.e., S_a_. In particular, the improvements in surface quality compared with that achieved with the Gaussian beam were 15% and 21% in case of the hatching (a) and hatching with outlining (c) strategies, respectively. There was only a small increase of surface roughness (3%) when the top-hat beam with the reduction outlining strategy (b) was used to produce the pockets. The uniform intensity distribution of the top-hat beam, especially the much reduce energy tail, led to a more even material ablation within the beam spot area and also there was less recast around the processed area. In addition, the drop of peak power when the Gaussian profile was transformed into a top-hat one while keeping the pulse energy the same led to a reduction of the average surface roughness. Roughness was improved with all three processing strategies while the average depth of the pocket decreased only marginally, in the range from 0.5% to 1.6%. This marginal decrease of the ablation rate can be compensated easily with small increases of pulse energy that should not have any impact on resulting roughness.

With both beam profiles, there was a tendency surface roughness to increase closer to pockets’ walls. This can be explained with the changes in the evacuation conditions for the ablated material and also due to some beam shadowing effects closer to the walls. Recast formation along the walls together with some processing debris could also be contributing to the high roughness.

The tapering effect in laser micro-processing is a major shortcoming, and minimizing and eliminating it is a challenge. The results in Table 3 clearly show that the top-hat processing had a beneficial effect on the taper angle along the pockets’ side walls. In particular, the taper angle was reduced by 2.3° (14.9%) and 0.7° (4.1%) in case of the hatching (a) and reduction outlining (b) strategies, respectively. However, the hatching with outlining (c) strategy with Gaussian beam led to a significantly smaller tapering effect along the side wall, i.e., approximately 50% less that the taper angle obtained with the top-hat profile. This could be explained with the high peak power of the Gaussian beam that led to higher ablation rates at the center of the beam spot. This led to a better material removal along the side walls, especially after the outlining passes along the walls.

**Figure 3:**
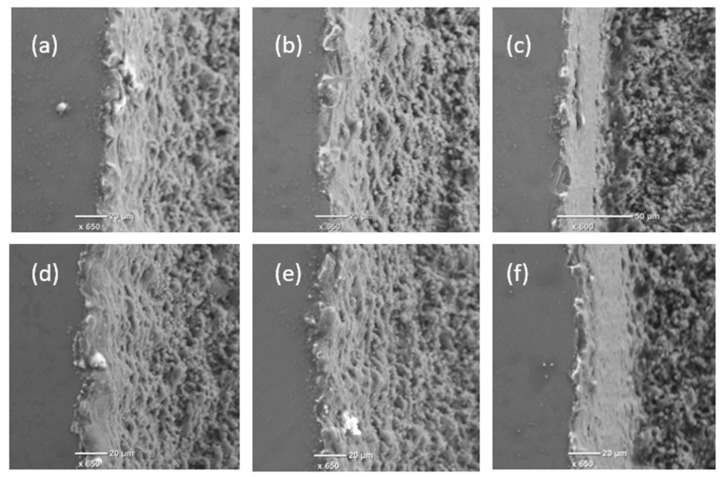
SEM images of the pocket edges produced with Gaussian (top) and top-hat (bottom) beams and the three strategies, respectively: Hatching (**a**,**d**), reduction outlining (**b**,**e**), and hatching with outlining (**c**,**f**).

The edge definition and HAZ are also important factors affecting the structuring quality that should be considered when comparing the pockets produced with both beam intensity profiles and the considered three processing strategies. Figure 3 depicts the edge quality achieved with both beam profiles and three processing strategies. The effect on HAZ cannot be seen clearly and the recast formations at the edges are quite similar for the structures produced with both beam profiles. As expected, distinctly cleaner and sharper edges were created with strategy (c) due to the additional outlining passes and the corrugated effect along the side wall (as shown in Figure 3a,b,d,e) was eliminated.

**Figure 4:**
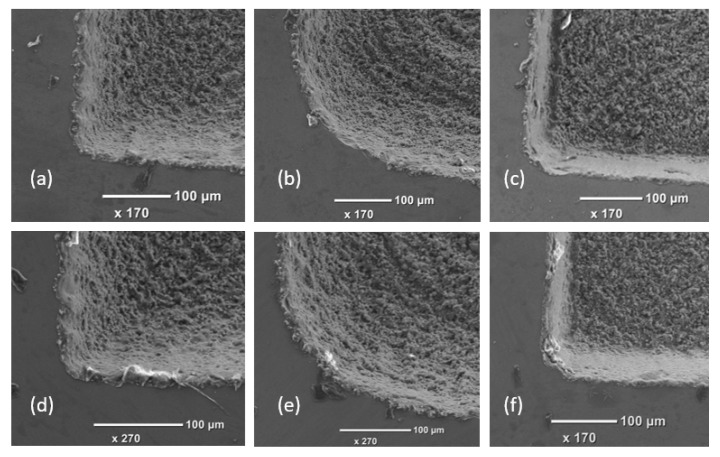
The sharpness of pocket corners achieved with Gaussian (top) and top-hat (bottom) beams and the three strategies, respectively: Hatching (**a**,**d**), reduction outlining (**b**,**e**), and hatching with outlining (**c**,**f**).

The sharpness of pocket corners was again strongly dependent on scanning strategy. Much better definition of the corner was achieved with hatching (a) and hatching with outlining (c) strategies compared with the reduction outlining one (b) as shown in Figure 4. The relatively poor corner definition resulting after strategy (b) can be attributed to some dynamic effects of the scan head at high speed when sharp changes in the beam movement directions are required. The effects of the top-hat processing on structuring quality are not as well pronounced as those on surface integrity. Especially, this is the case because such effects are highly dependent on the quality of the top-hat beam. The laser system used to conduct the experiments was not designed to integrate a beam shaper. Therefore, the quality of the top-hat beam achieved on this system might be affected by the employed manual procedures to retrofit and then to calibration the beam shaper. The beam shaper may work better on laser system, which is designed to install beam shaper, thus, to give better top-hat beam.

**Figure 5:**
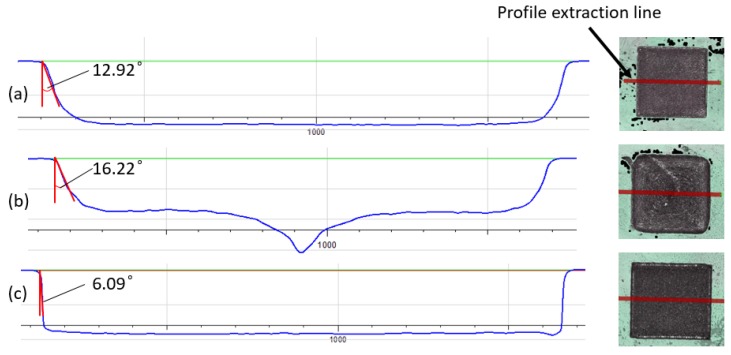
Representative cross sections of pockets produced with top-hat beam (pulse energy 0.15 mJ, hatch distance 15 μm, pulse distance 10 μm, and frequency 150 kHz) and the three strategies, respectively: (**a**) hatching, (**b**) reduction outlining, and (**c**) hatching and outlining.

The three investigated scanning strategies in this research had a clear impact on structure quality but also on their morphology. Cross-sectional views of the structures produced with the top-hat beam with these three strategies are presented in Figure 5. The best side walls in regard to the taper angle and the sharpness of the bottom corners were achieved with strategy (c) as was discussed above. In addition, the reduction outlining (b) strategy led to a significant deviation from the nominal pocket profile. In particular, the central part of the pocket was deeper as shown in Figure 5 because of the heat accumulation effect that led to high ablation rates there. This phenomenon occurs when laser passes are too close to each other. In addition, when outlining square trajectories become smaller at the center part, the scanner cannot reach the designated speed. Thereby, the actual overlap in center area is greater than the outer area. This partly contributed to higher ablation rate at center of the structure. However, it can be minimized by increasing pulse and hatching distances at central of the pocket. The accuracy of the pockets was much better when hatching (a) and hatching with outlining (c) strategies were used as shown in Figure 5.

#### 4.1.2. Process Efficiency

**Figure 6:**
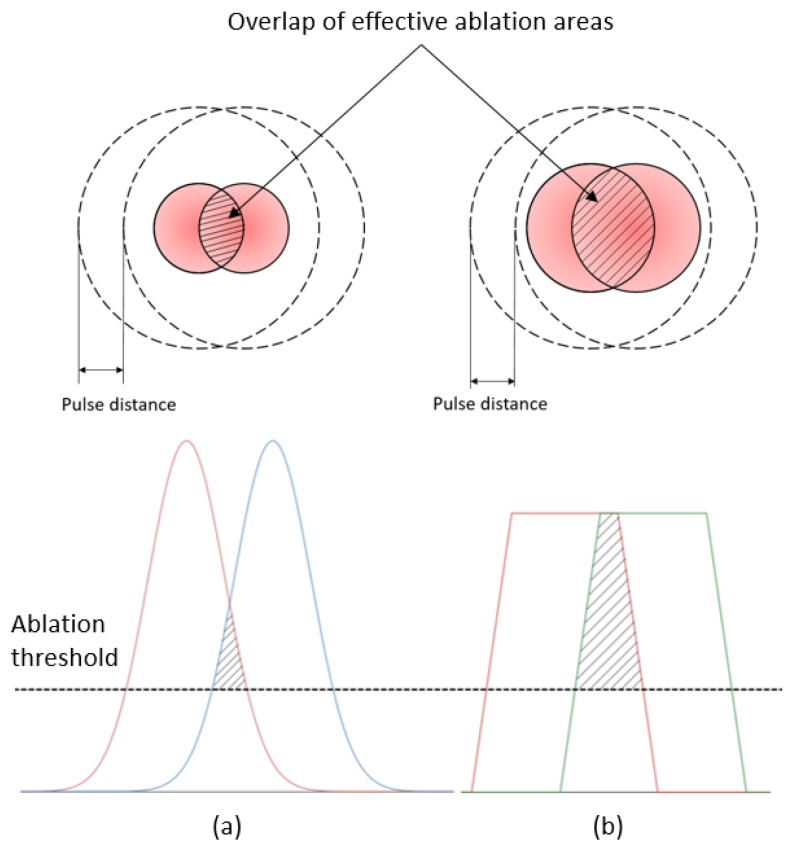
Overlaps of effective ablation areas achievable with Gaussian (**a**) and top-hat (**b**) beams with similar pulse and hatch distances.

The ablation rates were assessed based on the actual thickness of each processed layer and, thus, to compare the impact of beam profiles and also the respective three processing strategies (see Table 4). The ablation rates achieved with the top-hat beam were nearly equal (98% to 99.5%) to those achieved with Gaussian beam when the same pulse energy of 0.15 mJ was applied. The drop of peak power when structuring with top-hat beam led only to marginal reduction in ablation rates. Theoretically, the lower peak power of top-hat beams should lead to some reductions of ablation rates. However, at the same time the top-hat profile leads to bigger beam spot area with fluence levels higher than the ablation threshold compared to Gaussian one. Thus, larger overlaps of effective ablation areas can be achieved with the top-hat beam when pulse and hatch distances are similar as shown in Figure 6. Consequently, the heat accumulation phenomena affects a bigger area in case of top-hat processing where decreases ablation threshold [48] and, thus, leads to a better ablation efficiency. This compensates to larger extend the decrease of ablation rates in top-hat processing due to the drop of pulse peak intensity. It is evident from the experimental results that the heat accumulation effect can bring an important advantage in laser micro-processing, especially improvements in ablation rates can be achieved without sacrificing either structuring or surface quality.

Hatching with outlining (c) strategy led to the best taper angle and good average surface roughness (see Table 4). As stated in Section 4.1.1, outlining passes reduced the tapering effect but this was in expense of processing efficiency as they were additional to the hatching ones. It is important to note that the structuring efficiency, the achievable ablation rates, of silicon wafers is relatively good compare with some metals, e.g., stainless steel or copper-based alloys. Therefore, only five outlining passes were necessary after the hatching ones for each layer. However, if a material with lower ablation rates is used, e.g., stainless steel, the processing time will increase even further as more outlining passes will be required when hatching with outlining (c) strategy is employed. At the same time, this increase of processing time can be minimized due to bigger effective ablation areas achievable with top-hat profiles and also by increasing the pulse distance, e.g., by increasing the scanning speed and hatch distance. Therefore, the use of top-hat beams can expand the parameters’ domain that should be considered when optimizing the process, especially by taking into account the trade-offs between processing efficiency and structuring quality.

When the reduction outlining (b) strategy was used, the ablation depth per layer was lower, i.e., 94–95% lower, compared with other two strategies but at the same time the processing efficiency increased approximately three times even though the total beam path lengths were equal across the three strategies. The increase of processing time can be explained with the employed laser delays during the hatching passes that are necessary for achieving a good structuring accuracy while they are not necessary for the outlining passes in (b) strategy. However, as discussed before this high processing efficiency achieved with the reduction outlining strategy was in expense of structuring quality as discussed in Section 4.1.1. Thus, again it can be reiterated that there is a potential for further improvements of processing efficiency when top-hat beams are used by developing new structuring strategies that can combine the capabilities of hatching and outlining passes while taking into account the trade-offs associated with quality.

### 4.2. Structuring with Varying Hatching and Pulse Distances

**Figure 7:**
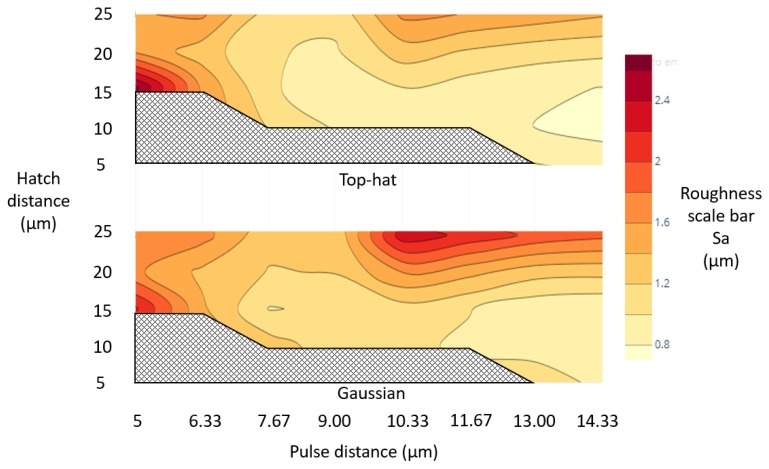
Roughness contour plots of pockets produced with top-hat and Gaussian beams and considered hatch and pulse distances (pulse length 65 ns, frequency 150 kHz, and pulse energy 1.5 mJ).

The effects of varying pulse and hatching distances with the hatching (a) strategy on resulting surface roughness and ablation rates were investigated. Contour plots that depicts the roughness levels achieved across the considered pulse and hatching distances with both beam profiles are given in Figure 7. The highest roughness was obtained when the pulse distance was in the range from 10.5 to 14.5 μm while the hatch distance was higher than 15 μm for both beam profiles. However, it should be mentioned that the increase of roughness with these process settings was less in case of the top-hat beam. The high roughness can be explained with relatively small pulse overlaps at these settings and as consequence of this the processing was not uniform while the heat accumulation was the relatively low, that also led to low processing efficiency. At the same time, the other extreme, i.e., too small pulse distances of less than 6 μm, also led to high roughness with both beam profiles. In this case, the heat accumulation was too much and led to more debris and recasts in the processed area.

Overall, the top-hat beam provided a lower roughness than the processing with Gaussian beam for most of investigated process settings. Thus, if a given roughness should be achieved, the processing with top-hat beam will offer more flexibility, especially processing with higher pulse and hatch distances, and, thus, a higher processing efficiency. Likewise, it should be stated that roughness lower than 0.8 μm was achieved with top-hat beam when the pockets were produced with pulse and hatch distances in the range from 13 to 14.5 μm and from 10 to 15 μm, respectively, while such lower roughness was not achieved with Gaussian beam. It is important to note that the hatch areas in Figure 7 depict regions where the accumulated laser fluence increased to such a level in the conducted experiments that it was sufficient to penetrate the workpiece.

### 4.3. Structuring with Varying Pulse Energies

**Figure 8:**
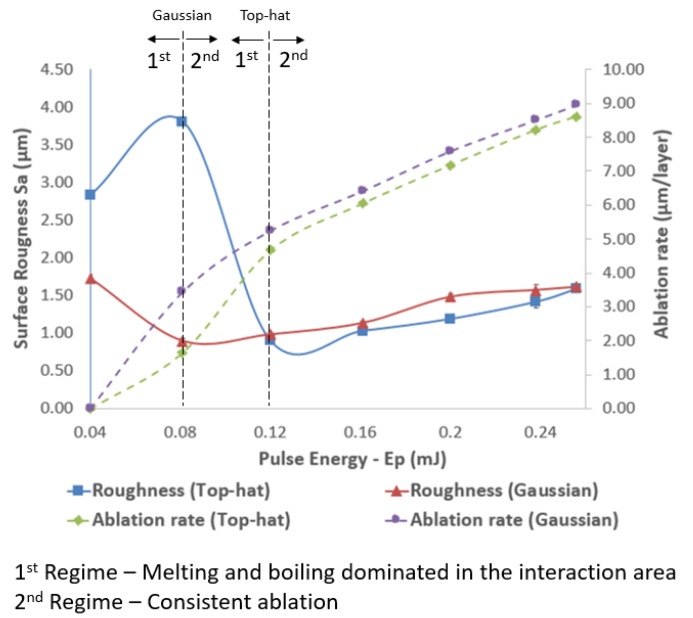
The effects of varying pulse energy on surface roughness and processing efficiency achievable with Gaussian and top-hat beams (pulse length 65 ns, frequency 150 kHz, pulse distance 10 μm, and hatch distance 15 μm).

The effects of varying pulse energies on structuring performance with both beam profiles were investigated, too. Especially, the impact of increasing pulse energies in the range from 0.04 to 0.256 mJ on resulting roughness and structuring efficiency is depicted in Figure 8. Two distinct processing regimes were identified when the effects of pulse energy on surface roughness were analyzed. The first regime was present when laser intensity was not sufficient and melting and boiling dominated in the interaction area. The second one was triggered when the intensity reached the required level for consistent ablation that led to a linear increase of material removal with the increase of pulse energy. The surface roughness was the highest, i.e., S_a_ of 1.7 μm, at the lowest pulse energy of 0.04 mJ, dropped to 0.9 μm at 0.08 mJ and then increased almost linearly to reach S_a_ of 1.6 μm at 0.256 mJ when the structuring was carried out with Gaussian beam.

A different surface response in regard to the resulting roughness was observed when the structuring was performed with a top-hat beam and the same range of pulse energies were investigated. Especially, roughness was S_a_ of 2.83 μm at the lowest pulse energy setting of 0.04 mJ and then initially increased to peak with S_a_ of 3.81 μm at *E*_p_ = 0.08 mJ. Next, roughness dropped to the lowest S_a_ value of 0.9 μm at 0.12 mJ and then again increased almost linearly to S_a_ of 1.6 at the highest pulse energy setting of 0.256 mJ. At the low pulse energy settings (first processing regime), the laser intensity was only sufficient for melting or partly boiling the substrate material that can explain the higher resulting roughness. The transition from first to second regime led to the lowest roughness that was achieved at different pulse energy levels for the two considered beam profiles, especially at lower setting for the Gaussian beam of 0.08 mJ while pulse energy of 0.12 mJ was required for the top-hat one.

The ablation rates at the lowest pulse energy settings of 0.04 mJ were zero for both Gaussian and top-hat beam. When the pulse energy increased to 0.08 mJ, there was some ablation with both Gaussian and top-hat beams, especially 3.44 and 1.65 μm/layer, respectively. The processing efficiency of the top-hat beam was less than half of that achieved with Gaussian beam while the Sa roughness values reached the two extremes, i.e., the highest (3.81 μm) and the lowest (0.89 μm), respectively. The high roughness values achieved with the top-hat beam can be explained with the relatively low peak power that was not sufficient to have a stable ablation process and therefore led to some recasts and melted material in the processed areas.

At the same time, when the pulse energy excessed 0.12 mJ, the structures fabricated with the top-hat beam had consistently a better surface roughness compared to that achieved with Gaussian beam. In particular, the improvements in surface roughness varied in the range of 1–2% at 0.256 mJ to almost 20% at 0.2 mJ when the pulse energy was varied from 0.12 mJ to 0.256 mJ. The average roughness improvement in this pulse energy range was 9.5%. Regarding the processing efficiency, the ablation rates achieved with the top-hat beam was 89% at 0.12 mJ to 97% at 0.238 mJ of the respective ones obtained with Gaussian beam. Thus, it can be stated that the ablation rates of top-hat beam were getting close to those achieved with Gaussian beam when pulse energy was increased. Thus, taking into account the bigger parameter domain that should be considered in optimizing the top-hat structuring process it should possible to achieve a better surface roughness with a comparable processing efficiency to that achieved with Gaussian beam.

## 5. Conclusions

The paper reports an investigation into the effects of beam intensity profiles and scanning strategies in laser micro-structuring. The resulting surface and structuring quality together with processing efficiency were compared when the other process parameters were kept the same. The result shows that the top-hat processing can lead to improvements both in surface roughness and structuring quality. Especially, the top-hat processing resulted into a decrease of the taper angle on the side walls of the structures in the range from 4% to 15% when only hatching and reduction outlining strategies were used. The tapering effect was smaller with Gaussian beam only when hatching with additional outlining passes were used. However, it is worth stressing that the improvements in surface roughness and the tapper angle achieved with the top-hat beam were not in expense of processing efficiency. In particular, the ablation rates were nearly the same with both beam intensity profiles or just marginally worst with the top-hat beam. The hatching strategy with some additional outlining passes resulted in a very good surface roughness and the lowest taper angle along the side walls with both beam profiles. The use of only outlining passes improved the processing efficiency almost three times but this was in expense of surface and structuring quality.

The effects of top-hat structuring when varying pulse energy and hatch and pulse distances were also investigated. The top-hat structuring led to a better surface and structuring quality when pulse energy higher than 0.12 mJ were used while the processing efficiency was almost the same. The dependence of surface quality on set hatch and pulse distances showed that a better surface quality in a bigger parameters’ domain can be achieved with a top-hat beam. In addition, a given pre-defined surface roughness can always be achieved with a better processing efficiency when the structuring is performed with a top-hat beam.

## Figures and Tables

**Table 1:** Laser processing parameters.

Parameter	Unit	Value
Power	W	22.5
Pulse energy	mJ	0.15
Pulse duration	ns	65
Frequency	kHz	150
Scanning speed	mm/s	1500
Number of layer		30

**Table 2:** Pulse and hatching distances and pulse energies used in the second and third sets of experiments.

Scanning Speed (mm/s)	750	950	1150	1350	1550	1750	1950	2150
**Equivalent pulse distance (µm)**	5.00	6.33	7.67	9.00	10.33	11.67	13.00	14.33
**Hatching distance (µm)**	5, 10, 15, 20, 25							
**Pulse energy (mJ)**	0.040	0.081	0.120	0.161	0.200	0.238	0.256	

**Table 3:** Inspection results of the structures produced with three processing strategies.

Processing Strategy	Gaussian			Top-Hat		
	Roughness S_a_	Depth	Taper Angle	Roughness S_a_	Depth	Taper Angle
	µm	µm	Degree	µm	µm	Degree
(1)	1.05 ± 0.05	187	15.19 ± 2.02	0.89 ± 0.02	184	12.92 ± 0.85
(2)	2.62 ± 0.09	175	16.92 ± 1.61	2.69 ± 0.08	174	16.22 ± 1.86
(3)	1.12 ± 0.05	186	3.02 ± 0.46	0.89 ± 0.02	185	6.09 ± 1.85

**Table 4:** Ablation rates and processing times achievable with the investigated 3 processing strategies.

Scanning Strategy	Ablation Rate (µm/layer)		Processing Time (30 layers) [s]
	Gaussian	Top-hat	
(a)	6.23	6.13	10.7
(b)	5.83	5.80	3.7
(c)	6.20	6.17	12.23
